# MedEval — A Swedish medical test collection with doctors and patients user groups

**DOI:** 10.1186/2041-1480-2-S3-S4

**Published:** 2011-07-14

**Authors:** Karin Friberg Heppin

**Affiliations:** 1 NLP-Unit, Department of Swedish, University of Gothenburg, S-405 30 Gothenburg, Sweden

## Abstract

**Background:**

Test collections for information retrieval are scarce. Domain specific test collections even more so, and medical test collections in the Swedish language non-existent prior to the making of the MedEval test collection. Most research in information retrieval has been performed in the English language, thus most test collections contain English documents. However, English is morphologically poor compared to many other European languages and a number of interesting and important aspects have not been investigated. Building a medical test collection in Swedish opens new research opportunities.

**Methods:**

This article describes the making of and potential uses of MedEval, a Swedish medical test collection with assessments, not only for topical relevance, but also for target reader group: Doctors or Patients. A user of the test collection may choose if she wishes to search in the Doctors or the Patients scenario where the topical relevance assessments have been adjusted with consideration to user group, or to search in a scenario which regards only topical relevance.

In addition to having three user groups, MedEval, in its present form, has two indexes, one where the terms are lemmatized and one where the terms are lemmatized and the compounds split and the constituents indexed together with the whole compound.

**Results:**

Differences discovered between the documents written for medical professionals and documents written for laypersons are presented. These differences may be utilized in further studies of retrieval of documents aimed at certain groups of readers. Differences between the groups of documents are, for example, that professional documents have a higher ratio of compounds, have a greater average word length and contain more multi-word expressions.

An experiment is described where the user scenarios have been utilized, searching with expert terms and lay terms, separately and in combination in the different scenarios. The tendency discovered is that the medical expert gets best results using expert terms and the lay person best results using lay terms, but also quite good results using expert terms or lay and expert terms in combination.

**Conclusions:**

The many features of MedEval gives a variety of research possibilities, such as comparing the effectiveness of search terms when it comes to retrieving documents aimed at the different user groups or to study the effect of compound decomposition in retrieval of documents. As Swedish, the language of MedEval, is a morphologically more complex language than English, it is possible to study additional aspects of the effect of natural language processing in information retrieval, for example utilizing different inflectional word forms in the retrieval of expert vs lay documents. MedEval is the first Swedish test collection of the medical domain.

**Availability:**

The Department of Swedish at the University of Gothenburg is in the process of making the MedEval test collection available to academic researchers.

## Background

Building a test collection is a major undertaking, therefore test collections are scarce. But the long process of building a test collection gives many insights in the field of information retrieval. This article describes the process from collecting the documents in the underlying corpus, through the creation of search topics, the instructions to the relevance judges including the choice of categories in the assessments of documents for relevance and for intended reader group. The article also presents the structure of the recall bases and the representation of the collection documents in the two indexes, with and without split compounds. To show how a test collection such as MedEval can be used, the article presents a selection of substantial differences between the documents written for professionals and documents written for laypersons, and finally presents experimental runs for the study of retrieval of documents aimed at the two target reader groups.

When the decision was made to build a new test collection, the Department of Swedish at the University of Gothenburg was involved in projects of research in medical language processing. There was also a growing interest of research in information retrieval. As no Swedish medical test collection existed, creating one seemed to be a good investment in knowledge and resources, even though this involved a team of people during many months.

One existing medical test collection, albeit in English, is OHSUMED [1]. It is built of nearly 350,000 references from MEDLINE, and thus the documents contained have medical professionals as intended readers. The OHSUMED documents are assessed on a three graded scale: definitely, possibly and not relevant. OHSUMED contains 106 topics generated by physicians from authentic situations. Each topic consists of information about a specific patient and an information request concerning this patient.

## Methods

### The collection documents

The MedEval test collection differs from OHSUMED in several ways. It is built on documents from the MedLex medical corpus [2] and contains documents intended both for medical professionals and for laypeople. MedLex consists of scientific articles from medical journals, teaching material, guidelines, patient FAQs, health care information, etc. The set of documents used in MedEval is a snapshot of MedLex in October 2007, approximately 42,200 documents or 15 million tokens (see Table 1). The documents are stored in the trectext format [3].

**Table 1:** The genres of the documents in the MedEval document collection

Type of source	Number of documents	Percent of documents	Number of tokens	Percent of tokens
Journals and periodicals	8,453	20.0	5.3 million	34.6
Specialized sites	14,631	34.6	2.9 million	19.1
Pharmaceutical companies	9,200	21.8	2.3 million	14.8
Government, faculties, institutes, and hospitals	2,955	7.0	2.0 million	13.3
Health-care communication companies	4,036	9.6	1.7 million	11.3
Media (TV, daily newspapers)	2,980	7.1	1.0 million	6.9

Total	42,255	100.1	15.2 million	100

The genres and sizes of the MedEval document sources. The MedEval document collection is a snapshot of the MedLex corpus in October 2007. (D. Kokkinakis, p.c.)

### Indexes

The MedEval test collection, in its present form, has two indexes. One where the documents are converted to lower case, tokenized and lemmatized, and one where the compounds also are decomposed. In the second index, the compound terms are indexed as a whole together with the compound constituents. For instance: the compound *saltkoncentration* (English: ‘salt concentration’) is indexed as *saltkoncentration*, *salt*, and *koncentration*. This makes it possible to find matches when a simplex term in a query is used in a document only as a compound constituent, or when a query contains a compound while a document only contains one or both of the corresponding simplex constituent terms. Dealing with compounds and their constituents is important in languages, such as Swedish, where the process of compounding is very productive. As the ratio of compounds in Swedish texts is around 10% (see Table 2) a major part of written information is stored in these compound terms [4].

**Table 2:** Type and token frequencies of terms

	Entire collection	Assessed documents	Doctors assessed	Patients assessed	Common files	Doctors relevant	Patients relevant
Number of documents	42,250	7,044	3,272	4,334	562	1,233	1,654
Tokens	12,991,157	5,034,323	3,232,772	2,431,160	629,609	1,361,700	988,236
Tokens/document	307	715	988	561	1,120	1,104	596
Average word length	5.75	6.04	6.29	5.73	6.16	6.33	5.63
Full form types	334,559	181,354	154,901	92,803	50,961	87,814	43,825
Lemma types	267,892	146,631	126,217	73,121	40,857	71,974	34,263
Lemma type token ratio	48.5	34.3	25.6	33.2	15.4	18.9	28.8
Compound tokens	1,273,874	573,625	412,475	237,267	76,117	179,580	92,420
Full form compound types	187,904	99,614	83,846	47,387	24,083	45,257	20,157
Lemma compound types	144,159	78,508	66,907	37,151	19,685	36,867	16,006
Ratio of compounds	0.098	0.114	0.128	0.098	0.120	0.132	0.094

Statistics for different categories of terms in different subsets of documents in the MedEval test collection.

### Topics

For the creation of the MedEval information needs, also called topics, two medical students in their fourth year of studies were hired. Their instructions were to create information needs that would be plausible in real medical situations, by doctors or by patients. Guided by explorative searches, the topic creators were asked to adjust the complexity of the topics so that the plausible number of relevant documents for each topic would be not less than five but still not much more than 50. 100 topics were created in the first stage. 62 of these were used in the collection. The process of creating topics was inspired by [5] and is described in more detail in [6].

**Figure 1:**
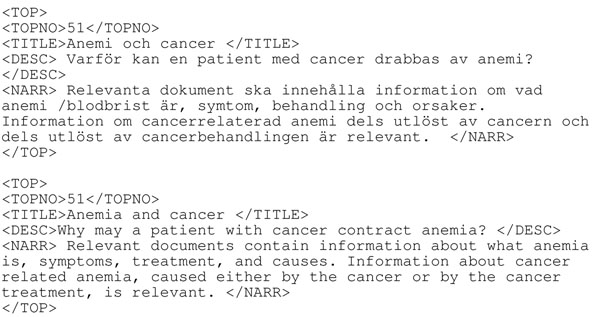
**Sample of information need**. An example of an information need, Topic 51, whith ID, title, description, and narrative. The information need is first given in Swedish, as in the collection, thereafter in an English translation.

A topic consists of a title, a description and a narrative. The title is a short phrase summarizing the information need. The description is concise information about the topic, usually in the form of a question or a request. The narrative is a few sentences long and it stipulates what makes a document relevant to the topic. The narrative contains the guidelines for the assessors when judging the relevance of the documents in the next stage. This conforms to the format of the TREC topics [3]. An example of a topic is given in Figure 1 together with an English translation.

### Selecting documents to assess

An ideal test collection would have a complete set of relevance judgments with every document assessed for relevance to every information need. With a collection of over 42,000 documents and with 62 information needs, as in MedEval, taking an estimated average of 8 minutes to assess each document, working 40 hours a week, it would take four persons over 42 years to finish the assessments.

Instead of assessing all documents for all topics, subsets of documents with a high probability of being relevant to each topic were extracted. These subsets were selected in a series of different runs using basic queries. Since there was limited time and economic resources creating MedEval, the extraction of documents was done on a small scale with only one search engine, namely Indri/Lemur [7].

Four different search methods were used in the extraction, that is, four runs for every information need. For each run, the 100 documents ranked most likely to be relevant were extracted, if in fact so many were retrieved. Two searches were done in each index: with and without decomposed compounds. One search was intended to be broad and one more specific. The number of documents assessed for each information need was between 115 and 358.

For each topic, the result of the extraction was four lists of document IDs. These were merged in one file per topic. The IDs were sorted in alphanumerical order and duplicates were removed. This is important to avoid bias, as the assessors must not know how the documents were ranked in the initial runs or in how many searches each document was retrieved. The documents corresponding to the extracted IDs were printed on paper and fixed in separate bundles for each topic. The papers were printed on only one side to avoid negative bias for short documents ending up on the left page of a spread. The method for selecting documents to assess was based on methods described in [3] and [8].

### Relevance judgments

The extracted documents were assessed for relevance according to the corresponding information needs. Four medical students were hired to do the assessments, not the same students as the creators of the MedEval topics. Domain knowledge is essential for understanding the topics and the contents of the documents and also for consistency in judging [9].

It may be expected that the greater the judges’ subject knowledge, the higher will be their agreement on relevance judgments. Subject knowledge seems to be the most important factor affecting the relevance judgment as far as human characteristics are concerned. [9], p. 341

For each of the 62 topics, an assessor read through the documents to be assessed and decided, for each document, the intended group of readers and the degree of relevance to the topic. The documents for each individual need were assessed by one and the same assessor for reasons of consistency. It is not unusual for assessors to disagree on the relevance of a certain document. However, considering two documents, assessors tend to agree which one is the more relevant. As research in information retrieval according to the Cranfield paradigm [10] is based on relative relevance scores, and not absolute relevance scores, this would make the judging sufficiently consistent [11]. This has been concluded in several studies, and already in [9].

It is most significant to note that the relative relevance score of documents in a group [...] may be expected to be remarkably consistent even when judges with differing backgrounds make the relevance judgments. Thus, it may be more profitable to compare the relative position of documents in a set than to compare the relevance ratings assigned to individual documents. [9], p. 341

The findings of Saracevic are supported by later studies conducted by Voorhees [12]. She claims that the important question is not how well assessors agree with one another, but how the results change with these differences in assessement. Her conclusion is that despite differences in assessments between assessors, the evalutation behavior remains the same. Supported by [11], [9], and [12] the creators of the MedEval test collection came to the conclusion that one assessor per topic would be sufficient. More important for obtaining a consistent test collection was to not split any set of documents assessed for a certain topic between different assessors.

The MedEval relevance assessments were made on a four graded scale, 0–3, where 0 is ‘Not at all relevant’ and 3 is ‘Highly relevant’ [13]. This scale is easily turned into a binary scale by stating that the documents with the lower grades are to be considered non-relevant and the ones with higher grades relevant. An impatient user, who is satisfied with one or a few documents, could have only documents with relevance score 3 considered relevant, while a user who is willing to take her time, and who wants as many documents as possible, could let all documents with relevance 1–3 be considered relevant.

The relevance judged by the assessors was topical relevance, how well a document corresponds to a topic. The assessors were instructed not to involve user relevance in this score. Each document was judged on its own merits. The novelty of the contents of a document should not be taken into account.

### Target groups

In addition to topical relevance the assessors judged each document for target reader group, that is which group of readers was the intended: Patients, if a document was written for laypersons, or Doctors, if it was written for medical professionals. This assessment was not based on any statistical or formal factors, only on the assessors’ judgments. Some documents were difficult to classify as they were not clearly aimed at a certain group. A number of these documents were labeled with different target groups when assessed for different topics (see Table 2).

For a classification of documents according to intended reader group to be useful, there must be a measureable difference between the document classes. Table 2 shows statistics for different categories of terms in different subsets of the collection. In each set, duplicates were removed in the case that a document had been assessed for more than one topic. The subsets considered are described below. Full form types are the original terms of the documents before lemmatization (with inflections) and lemma types are the same terms after lemmatization (reduced to base form).

**Entire collection** All documents of the MedEval collection.

**Assessed documents** All documents that have been assessed for any topic.

**Doctors assessed** All documents that for at least one topic have been assessed to have target group Doctors.

**Patients assessed** All documents that for at least one topic have been assessed to have target group Patients.

**Common files** All documents that for at least one topic have been assessed to have target group Doctors and for another to have target group Patients.

**Doctors relevant** All documents that for at least one topic have been assessed to have at least relevance grade 1 and to have target group Doctors.

**Patients relevant** All documents that for at least one topic have been assessed to have at least relevance grade 1 and to have target group Patients.

Before counting frequencies, the files were cleaned from tags, IDs, dates (in the date tag, not in the actual text), web information and punctuation marks. As the tokens were counted after the cleaning of the text, the number of tokens in this table is not consistent with the number of tokens in Table 1.

The number of tokens per document is significantly smaller for the entire collection, than for any subset. This means that there is a large number of short documents that were not retrieved by any query when the documents were extracted. This is not surprising, since short documents contain few terms which can match the queries. The finding that unjudged documents on average tend to be shorter than judged documents, both relevant and non-relevant, is consistent with the results of experiments described in [14]. One reason, according to Karlgren, is that non-retrieved items often contain tables and numerical information. He also concludes that longer documents have a bigger chance of touching relevant subjects, but unfortunately also confusingly similar subjects which are non-relevant.

The documents in the set ‘Patients assessed’ had only 57% the number of tokens per document, compared to the documents in ‘Doctors assessed’. Even though there were over 1,000 more documents in ‘Patients assessed’ than in ‘Doctors assessed’, there were over 50,000 more lemma types in the doctor documents and almost 30,000 more lemma compound types. Type token ratio is a measure of the average times each type, or word form, is used. This measure grows as the size of the set of documents considered grows. This fact makes it even more noteworthy that the type token ratio for the patient documents is significantly higher than for the doctor documents, even though the doctor documents contain more tokens. What this signifies is that there are not as many different types of word forms in the lay texts, but each type is used a larger number of times.

The average word length in ‘Doctors assessed’ was 6.29 compared to 5.73 for ‘Patients assessed’. The ratio of compound tokens was also higher in the doctor documents, 0.128 compared to 0.098.

Additional file 1 illustrates the fact that the doctor documents contain more and longer terms and more compounds than patient documents. The file shows frequencies of all full form types of strings beginning with the random term *förmak* ‘atrium’ in ‘Patients assessed’ and ‘Doctors assessed’ respectively. The patient documents have 18 full form types beginning with *förmak* while doctor documents have 75, more than four times as many.

Looking at all instances of strings beginning with *förmak* in the two sets of documents, for professional and laypeople, there is a significant difference. In the patient documents 66 tokens of 372, or 17.7%, are nouns in the definite form, while the corresponding numbers for the doctor documents is 89 of 932 tokens, or 9.6%. A hypothesis for why this is so, is that medical professionals often discuss matters in a generic point of view, while laypeople discuss specific cases.

Not only Swedish nouns, but also adjectives are inflected for definiteness and number. When comparing the word forms of adjectives in the doctor and patient documents, it is evident that the indefinite non-neuter singular form has relatively higher frequencies in the patient documents. This form would be the one a patient uses when speaking about him or herself or the doctor would use when addressing a patient, but also the form a physician would use when describing ‘a patient’. The definite and the plural adjective inflectional forms are identical to each other, but differ from the indefinite singular forms for most adjectives. This form is used for example when talking about ‘the patient’, ‘the patients’ or ‘patients’. Table 3 shows how the frequencies differ for a few adjectives.

**Table 3:** Frequencies of adjectives

		Doctor documents		Patient documents	
Term	Equivalent	Non-neuter singular indefinite	Plural and/or definite	Non-neuter singular indefinite	Plural and/or definite

sjuk	sick	165	462	333	371
smittad	infected	115	501	332	320
fet	fat	67	137	219	193
tjock	thick/fat	59	15	152	28
smal	thin	22	21	41	25
gravid	pregnant	78	471	651	402
allergisk	allergic	364	210	432	282
överkänslig	hypersensitive	15	10	72	15
deprimerad	depressed	20	89	79	42

Adjectives in non-expert documents have a stronger tendency to be in the singular indefinite form than adjectives in the expert documents. This corresponds to the patient documents having a tendency of being interactive in their approach while doctor documents often describe generic cases.

The conclusion that professionals discuss generic cases while laypeople discuss specific cases is supported by a difference that can be seen in frequency tables of multi-word expressions in doctor and patient documents [6]. High frequencies are found for phrases with meanings such as: *in patients with* or *of patients with.* Frequencies are also high for indefinite noun phrases such as: *in treatment of* or *for treatment of* The patient documents, on the other hand, contain phrases describing specific patients or specific cases, for example phrases that contain the pronoun *you* and noun phrases in definite form: *when the treatment is completed.*

Overall, the documents written for the doctor target group tend to be written in a more disassociated way compared with the patient documents which are more interactive in their approach, addressing the reader directly. While the professional documents tend to discuss research results or cases in general, the lay documents often discuss specific cases. This difference in approach manifests itself, for example in the features described above with the patient documents containing more nouns and adjectives in the definite form, and more pronouns in the first or second person, while doctor documents predominately have nouns and adjectives in the indefinite form, and pronouns in the third person. The professional documents also tend to be written in a more formal way with many multi-word phrases recurring with high frequencies. As there is an apparent difference between the documents written for the professional and layperson target groups, these differences could be used for a precategorization of documents according to genre. Such a categorization could be stored in a separate field in the document representations.

An interesting research question for future projects could be to study the benefit of lemmatizing inflected words, but keeping the inflectional information in tags, or recording the tendency of a text in terms of generic vs specific. This could be a way to keep the higher recall gained by lemmatization, but still use inflectional information for discrimination [6].

### User groups

The MedEval test collection allows the user to state user group: *None* (no specified group), *Doctors* or *Patients*. This choice directs the user to one of three scenarios. The None scenario contains the topical relevance grades as made by the assessors. The Doctors scenario contains the same grades with the exception that the grades of the documents marked for Patients target group are downgraded by one. In the same way the Patients scenario has the documents marked for Doctors target group downgraded by one. This means that for a doctor user patient documents by the assessor given relevance 3, are graded with 2, documents given relevance 2 are graded 1 and documents given relevance 1 are graded 0. The same is done in the Patients scenario with the doctor documents. The idea is that a document that is written for a reader from one target group but retrieved for a user from the other group will not be non-relevant, but less useful than a document from the correct target group. Put differently, a document intended for patients would contain information that doctors (hopefully) already know. On the other hand, documents intended for doctors, even though they might be topically relevant for a patient’s need, run a great risk of being written in such a way that a patient will have problems grasping the whole content. This is a way of introducing utility without performing user studies.

**Figure 2:**
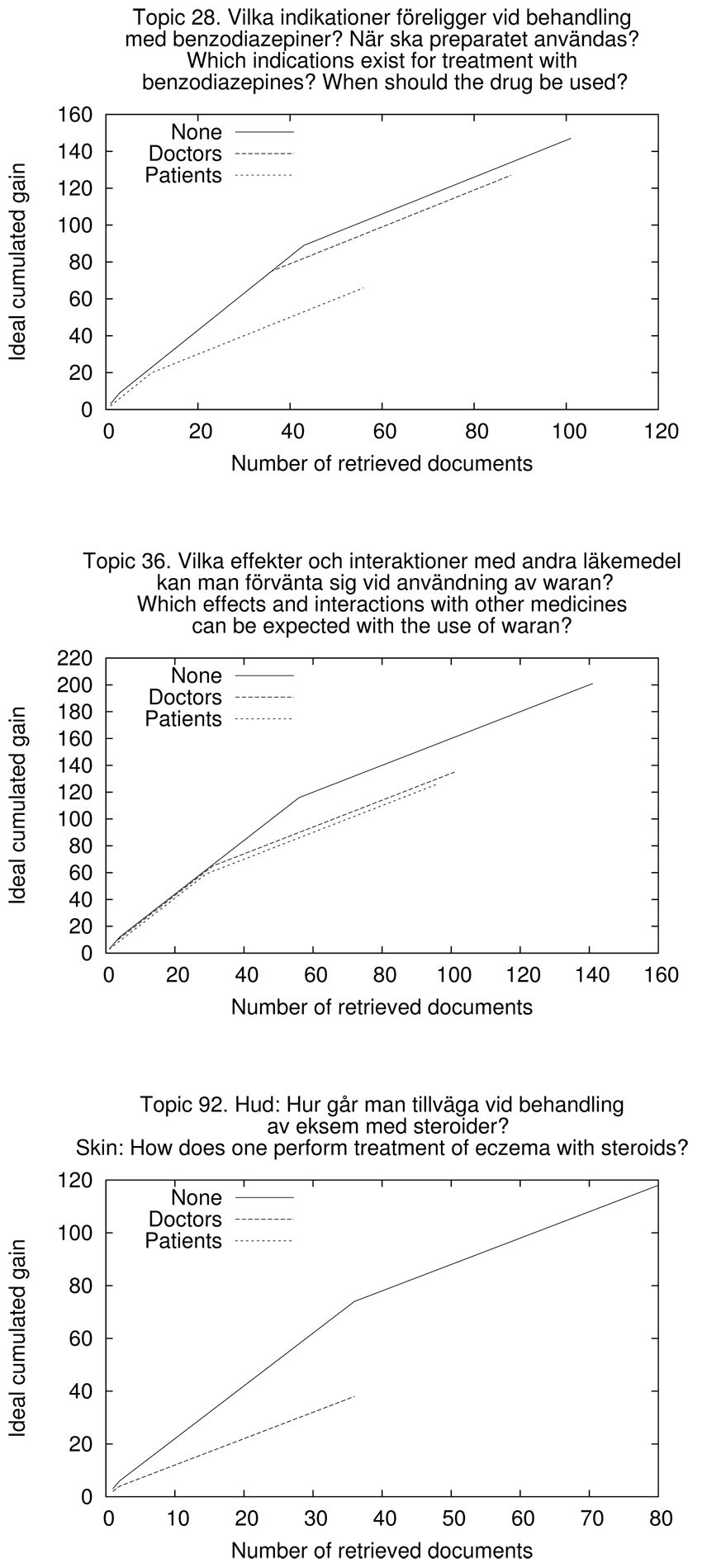
**Recall bases for three topics and three scenarios**. The recall bases of Topics 28, 36, and 92 represented in ideal cumulated gain for the three scenarios: None, Doctors and Patients. For Topic 28 most of the highly relevant and fairly relevant documents were assessed to have target group Doctors. Topic 36 had the relevant documents spread fairly evenly between the Doctors and Patients target groups. Topic 92 showed no documents of any relevance grade for documents marked for target group Doctors. Thus the None and the Patients ideal gain vector coincide fully, while the cumulated gain for the Doctors scenario is very low originating from the downgraded patient documents.

Adjusting relevance in the manner described affects the scenario recall bases. Since relevance grades are downgraded for documents of the opposing target group there will be fewer relevant documents in the Doctors and Patients scenarios than in the None scenario. This is demonstrated in Figure 2 where the ideal cumulated gain for the three scenarios of Topics 28, 36, and 92 are shown. The ideal cumulated gain is the maximum score of retrieved information possible at each position in a ranked list of documents [15]. The score for each position is the sum of all relevance scores so far in the ranked list.

The three topics of Figure 2 show different characteristics with reference to the number of relevant doctor and patient documents. Topic 36 has fairly similar cumulated gain curves for the Doctors and Patients scenarios. Topic 28 has a majority of doctor documents, while Topic 92 has no documents of any relevance grade for documents marked for target group Doctors. Thus the None and the Patients ideal gain vector coincide fully, while the cumulated gain for the Doctors scenario is very low, originating from downgraded patient documents.

## Results

To demonstrate the effectiveness of search terms from the different styles of language of the two target groups, a number of synonym pairs were used as search keys for corresponding topics. Each synonym pair consisted of one neoclassical term, belonging to the expert register, and one lay term. The terms of each pair were run separately as single search key queries, and also combined in one query. All queries, three for each topic, were run in the doctors scenario and in the patients scenario. Note that for each query the resulting ranked list of documents is the same for both scenarios. It is the recall bases, and thus the relevance grades of the retrieved documents, that differ.

As MedEval, to the authors’ knowledge, is the first medical test collection with user groups, there are no earlier equivalent tests. However, [16] address the fact that medical experts and non-experts express themselves in different ways, and that this affects search results. The authors are motivated by the empowerment of laypersons and discuss how to exchange information across user groups. The goal is that a search using non-expert terms should retrieve all types of documents written on the topic. They see the problem as a question of automatic alignment between specialized terminology and general terminology and enrich the information retrieval system with a set of links between corresponding concepts in lay and professional language.

The contrast between Swedish professional medical language and Swedish lay language is addressed in [17]. The authors have selected documents concerning cardiovascular disorders from the MedLex corpus [2]. Their findings may be used as a basis for future studies on how to differ searches with the purpose of retrieving documents for the different user groups. The findings have inspired the choice of entries in Table 2 showing differences between the sets of doctor and of patient documents.

### Measures of effectiveness

The effectiveness of the queries described above was measured in recall after 10, 20, and 100 retrieved documents. This represents the impatient, the slightly less impatient, and the patient user. The effectiveness was also measured in normalized discounted cumulated gain, nDCG [15]. The nDCG is based on the cumulated gain described earlier, but uses a discounting factor which reduces the amount of the relevance score added for each document in the ranked list. The relevance score is discounted by a logarithmic function of the position number. The assumption is that the later in the list a document is found, the less it is worth to the user. The normalization infers that the discounted cumulated gain is compared to the ideal discounted cumulated gain in each position. Thus the nDCG value summarizes the effectiveness in all positions earlier in the ranked list, and compares this summarized effectiveness to the maximum value possible in each position. As the nDCG value is relative to the maximum value possible, it varies between 0 and 1 and gives no bias to topics with small or large recall bases.

Even though recall and nDCG both measure effectiveness, there is not an absolute correlation between them. Recall is calculated on a binary scale. In this case documents with relevance score 1 are considered non-relevant. The nDCG, on the other hand is calculated on a four-graded scale, 0-3, and all scores from 1 to 3 are included in the measure. This entails that the nDCG value can seem high compared to the recall value if the ranked list includes documents with relevance score 1. On the other hand the recall value can seem high compared to the nDCG value if there are relevant documents late in the ranked list.

### The runs

Two of the more striking results are the runs for Topics 51 and 66, shown in Tables 4 and 5. For both of these topics the lay terms have very low effectiveness in the doctors scenario. There is no gain in using the lay term, neither as a single search key nor in combination with the neoclassical term. In the patients scenario there is less difference between the expert and the lay terms, used as single search key queries. The best result is achieved by using both terms in combination.

**Table 4:** Runs for Topic 51

	Effectiveness	anemi ‘anemia’	blodbrist ‘anemia’	Both
**Topic 51**	Recall@10	50% (4/8)	0% (0/8)	0% (0/8)
**Doctors**	Recall@20	87% (7/8)	0% (0/8)	0% (0/8)
**Scenario**	Recall@100	100% (8/8)	0% (0/8)	100% (8/8)
	nDCG@100	0.77	0.25	0.48

**Topic 51**	Recall@10	28% (5/18)	33% (6/18)	33% (6/18)
**Patients**	Recall@20	39% (7/18)	39% (7/18)	50% (9/18)
**Scenario**	Recall@100	72% (13/18)	56% (10/18)	89% (16/18)
	nDCG@100	0.60	0.61	0.76

Varför kan en patient med cancer drabbas av anemi?

Why may a patient with cancer contract anemia?

**Table 5:** Runs for Topic 66

	Effectiveness	anafylaxi ‘anaphylaxis’	allergisk chock ‘allergic shock’	Both
**Topic 66**	Recall@10	43% (3/7)	0% (0/7)	29% (2/7)
**Doctors**	Recall@20	57% (4/7)	0% (0/7)	43% (3/7)
**Scenario**	Recall@100	57% (4/7)	0% (0/7)	57% (4/7)
	nDCG@100	0.66	0.03	0.53

**Topic 66**	Recall@10	67% (2/3)	0% (0/3)	33% (1/3)
**Patients**	Recall@20	67% (2/3)	33% (1/3)	100% (3/3)
**Scenario**	Recall@100	67% (2/3)	33% (1/3)	100% (3/3)
	nDCG@100	0.50	0.19	0.55

Hur behandlas anafylaxi till följd av allergi?

How is anaphylaxis due to allergy treated?

Topic 63, in Table 6, shows low recall for the lay term in the doctors scenario as well as low recall and nDCG for the expert term in the patients scenario. In the doctors scenario the recall does not improve by adding the lay term to the expert term query. However the nDCG value improves. This means that relevant documents now appear earlier in the list. In fact the nDCG value for the lay term is surprisingly high. A closer look at the ranked list shows not less than six documents with relevance score 1 among the first ten, giving a high nDCG value. For this topic there is no gain in the patient scenario in combining the two terms, most likely because the neoclassical term has very low effectiveness.

**Table 6:** Runs for Topic 63

	Effectiveness	ventrikel ‘stomach’	magsäck ‘stomach’	Both
**Topic 63**	Recall@10	50% (2/4)	0% (0/4)	50% (2/4)
**Doctors**	Recall@20	50% (2/4)	0% (0/4)	50% (2/4)
**Scenario**	Recall@100	50% (2/4)	50% (2/4)	50% (2/4)
	nDCG@100	0.30	0.39	0.45

**Topic 63**	Recall@10	0% (0/6)	50% (3/6)	0% (0/6)
**Patients**	Recall@20	0% (0/6)	67% (4/6)	17% (1/6)
**Scenario**	Recall@100	0% (0/6)	83% (5/6)	67% (4/6)
	nDCG@100	0.12	0.55	0.35

Vilka tekniker och redskap används vid biopsi av magsäck vid cancermisstanke?

What techniques and equipment are used when performing biopsy of the stomach suspecting cancer?

For Topic 48, in Table 7, we again see low results for the lay term in the doctors scenario, while there is less difference between the terms in the patient scenario. In both scenarios we see a significant improvement when the two terms are used in combination.

**Table 7:** Runs for Topic 48

	Effectiveness	esofagus ‘esophagus’	matstrupe ‘esophagus’	Both
**Topic 48**	Recall@10	12% (2/16)	0% (0/16)	12% (2/16)
**Doctors**	Recall@20	25% (4/16)	0% (0/16)	19% (3/16)
**Scenario**	Recall@100	50% (8/16)	19% (3/16)	56% (9/16)
	nDCG@100	0.30	0.13	0.46

**Topic 48**	Recall@10	0% (0/7)	0% (0/7)	29% (2/7)
**Patients**	Recall@20	29% (2/7)	14% (1/7)	29% (2/7)
**Scenario**	Recall@100	29% (2/7)	57% (4/7)	57% (4/7)
	nDCG@100	0.23	0.23	0.53

Topic 48. *Vad är prognosen vid olika typer av cancer i matstrupen?*

What is the prognosis of various types of cancer of the esophagus?

For Topic 7, Table 8, the neoclassical term gives best results in both the doctors and the patient scenarios, while for Topic 83, Table 9, the lay term gives best results in both cases. The patient scenario for Topic 7 does not show any gain in combining the search keys, the best result is still using the expert term. Topic 83, which has an effective lay term, even in the doctors scenario, here shows improved effectiveness when combining the terms.

**Table 8:** Runs for Topic 7

	Effectiveness	cytostatika ‘chemotherapy’	cellgift ‘chemo’	Both
**Topic 7**	Recall@10	19% (5/27)	15% (4/27)	7% (2/27)
**Doctors**	Recall@20	30% (8/27)	19% (5/27)	7% (2/27)
**Scenario**	Recall@100	52% (14/27)	33% (9/27)	37% (10/27)
	nDCG@100	0.54	0.28	0.28

**Topic 7**	Recall@10	17% (8/47)	6% (3/47)	4% (2/47)
**Patients**	Recall@20	23% (11/47)	11% (5/47)	13% (6/47)
**Scenario**	Recall@100	70% (33/47)	15% (7/47)	30% (14/47)
	nDCG@100	0.60	0.29	0.33

Vilka biverkningar kan man räkna med vid behandling av cancer med cellgift?

Which side effects can one expect when treating cancer with chemotherapy?

**Table 9:** Runs for Topic 83

	Effectiveness	synkope ‘syncope’	svimning ‘fainting’	Both
**Topic 83**	Recall@10	43% (3/7)	43% (3/7)	43% (3/7)
**Doctors**	Recall@20	43% (3/7)	43% (3/7)	57% (4/7)
**Scenario**	Recall@100	43% (3/7)	57% (4/7)	57% (4/7)
	nDCG@100	0.39	0.47	0.57

**Topic 83**	Recall@10	20% (2/10)	50% (5/10)	50% (5/10)
**Patients**	Recall@20	20% (2/10)	50% (5/10)	60% (6/10)
**Scenario**	Recall@100	20% (2/10)	60% (6/10)	60% (6/10)
	nDCG@100	0.19	0.53	0.48

Vilka är de bakomliggande orsakerna till synkope och hur behandlar man det?

What are the underlying causes of syncope and how is it treated?

Topic 68, in Table 10, has reasonable results for all single search key queries in both scenarios. The best results are for the expert term in the doctors scenario and for the lay term in the patients scenario. In both scenarios the recall is improved when the terms are combined, but the nDCG value is not.

**Table 10:** Runs for Topic 68

	Effectiveness	trombos ‘thrombosis’	blodpropp ‘blood clot’	Both
**Topic 68**	Recall@10	18% (6/34)	6% (2/34)	9% (3/34)
**Doctors**	Recall@20	21% (7/34)	12% (4/34)	15% (5/34)
**Scenario**	Recall@100	56% (19/34)	29% (10/34)	68% (23/34)
	nDCG@100	0.51	0.33	0.48

**Topic 68**	Recall@10	18% (3/17)	24% (4/17)	18% (3/17)
**Patients**	Recall@20	24% (4/17)	41% (7/17)	24% (4/17)
**Scenario**	Recall@100	35% (6/17)	65% (11/17)	82% (14/17)
	nDCG@100	0.37	0.62	0.56

Vilka symtom associeras med DVT, djup ventrombos, och hur ser behandlingen ut?

Which are the symptoms associated with DVT, deep venous thrombosis, and what does the treatment look like?

In most cases, and not surprising, the expert terms are most effective in the doctors scenario and the lay terms in the patient scenario, but there are both expert and lay terms that achieve best results in both scenarios. The expert terms tend to give better results in the patient scenario than the lay terms in the doctors scenario. However more extensive studies, including comparisons of search results with relative frequencies of lay and expert terms, are needed before definite conclusions can be drawn.

## Conclusions

This article describes the process of building a test collection for information retrieval purposes. The process includes the collection of a corpus, creation of search topics, decisions about relevance assessments, such as selecting documents to assess and deciding on the assessment categories for the judges. Further the process includes how to represent the recall bases and how to represent the documents in the collection indexes.

The article goes on to show a number of aspects of medical information retrieval which can be studied utilizing the MedEval test collection. The main novelty of the collection is the marking of document target groups, Doctors and Patients, together with the possibility to choose user group. This opens for new areas of research in Swedish information retrieval such as how one can retrieve documents suited for different groups of users. As was shown in the example runs, search keys from different registers behave differently in the doctors and in the patients scenario.

A number of differences between the documents written for experts and for non-experts are presented along with the suggestion that these differences may be utilized in future studies of document retrieval for the different user groups.

Not least important is that MedEval is a Swedish domain specific test collection. A test collection in a language other than English allows a new range of research possibilities studying the impact of natural language processing in information retrieval.

The Department of Swedish at the University of Gothenburg is in the process of making the MedEval test collection available to academic researchers.

## List of abbreviations used

FAQ: Frequently Asked Questions; nDCG: normalized Discounted Cumulated Gain.

## Competing interests

The author declares that she has no competing interests.

## Authors contributions

KFH conceived of the study, supervised the building of the MedEval test collection, carried out the experiments and data analysis as well as manuscript preparation.

## Supplementary Material

Additional file 1 **Types and tokens in the doctor and in the patient documents** The file presents a randomly chosen example illustrating the difference in the number of types and the number of tokens for each type in the documents written for a lay audience and of the ones written for a professional audience. The table shows all types and frequencies of types of strings beginning with the string *förmak* ‘atrium’ in the two sets of documents. Click here for file
