# Supplementary material for: MedEval — A Swedish medical test collection with doctors and patients user groups
Source: J Biomed Semantics. 2011 Jul 14;2(Suppl 3):S4. doi: 10.1186/2041-1480-2-S3-S4 (PMC3194176; doi:10.1186/2041-1480-2-S3-S4)
Supplement: Additional file 1 — Types and tokens in the doctor and in the patient documents The file presents a randomly chosen example illustrating the difference in the number of types and the number of tokens for each type in the documents written for a lay audience and of the ones written for a professional audience. The table shows all types and frequencies of types of strings beginning with the string förmak ‘atrium’ in the two sets of documents. [file 2041-1480-2-S3-S4-S1.pdf]

Types and tokens in the doctor and in the patient documents

This is a randomly chosen example showing the difference in the number of types and the number of tokens for each type in the documents written for a lay audience and of the ones written for a professional audience. The table compares all types and frequencies of types of strings beginning with *förmak* 'atrium' in the two sets of documents. The strings in bold are misspelled.

**Professional audience**		**Lay audience**	
förmak	93	förmak	73
förmaken	21	förmaken	21
förmakens	1	förmakens	2
förmaket	11	förmaket	14
förmakets	1		
förmaks	21	förmaks	1
förmaksaktivering	1		
förmaksaktivitet	1		
förmaksaktiviteten	2		
förmaksanatomi	1		
förmaksarytmi	2		
förmaksarytmier	9	förmaksarytmier	2
förmaksbidraget	1		
förmaksbradyarytmi	1		
förmaksdefibrillator	2		
förmakseffekt	2	förmakseffekt	1
förmaksfladder	57	förmaksfladder	2
förmaksfladdret	2		
		**förmaksflimer**	1
förmaksflimmer	544	förmaksflimmer	219
förmaksflimmerablationer	2		
förmaksflimmerattacker	1	förmaksflimmerattacker	1
förmaksflimmerduration	2		
förmaksflimmerepisoder	4		
förmaksflimmerfladder	2		
förmaksflimmerpatienter	4	förmaksflimmerpatienter	1
förmaksflimmerrecidiv	1		
förmaksflimmertendensen	1		
förmaksflimmerunderhållande	1		
förmaksflimret	16	förmaksflimret	28
förmaksflimrets	4		
förmaksfrekvenser	1		
förmaksfunktion	1		
förmaksförstoring	1		
förmaksimpuls	1		
förmaksinhiberad	1		
förmakskontraktion	4		
förmakskontraktionen	6		
förmakskontraktionens	1		
		**förmakslimmer**	1
förmaksmuskeln	1		

Continued on next page...

...continued from previous page.

**Professional audience**		**Lay audience**	
förmaksmuskelns	1		
förmaksmuskulaturen	2		
förmaksmyocyterna	2	förmaksmyocyterna	1
förmaksmyokard	3		
förmaksmyokardiet	1		
förmaksmyxom	2		
förmaksnivå	2		
förmaksnära	1		
**förmaksoch**	1		
förmakspacing	7		
förmakspeptider	1		
förmaksrytmer	1		
förmaksseptostomi	1		
förmaksseptum	2		
förmaksseptumaneurysm	10		
förmaksseptumdefekt	5		
förmaksseptumdefekten	1		
förmaksseptumdefekter	1		
förmaksseptums	1		
förmaksstimulerat	1		
förmaksstimulering	5		
förmaksstorlek	2		
förmaksstorleken	1		
förmakssynkron	1		
förmakssystole	1		
förmakstaket	1		
förmakstakykardi	11	förmakstakykardi	1
förmakstakykardie	8		
förmakstromb	2		
förmakstryck	1		
förmakstrycket	1		
		förmaksutlösta	2
förmaksvolym	2		
förmaksvägg	1		
förmaksväggarna	2		
förmaksväggen	6		
förmaksvävnaden	2		
förmaksöra	9	förmaksöra	1
förmaksöronen	2		
